# Functional recovery after cesarean delivery: a prospective cohort study in rural Rwanda

**DOI:** 10.1186/s12884-023-06159-3

**Published:** 2023-12-13

**Authors:** Anne Niyigena, Saidath Gato, Barnabas Alayande, Elizabeth Miranda, Bethany Hedt-Gauthier, Andrea S. Goodman, Theoneste Nkurunziza, Christian Mazimpaka, Sadoscar Hakizimana, Patient Ngamije, Fredrick Kateera, Robert Riviello, Adeline A. Boatin

**Affiliations:** 1 Partners In Health/Inshuti Mu Buzima, KG 9 Avenue 46, PO Box 3432, Remera, Kigali, Rwanda; 2 University of Global Health Equity, Kigali, Rwanda; 3 Harvard Medical School, Boston, MA USA; 4 Technical University of Munich, Munich, Germany; 5 Kirehe District Hospital, Ministry of Health, Kirehe, Rwanda; 6 Brigham and Women’s Hospital, Boston, MA USA; 7 Massachusetts General Hospital, Boston, MA USA

**Keywords:** Cesarean section, functional recovery, rural Rwanda, resumption of activities, postoperative care

## Abstract

**Introduction:**

Women who deliver via cesarean section (c-section) experience short- and long-term disability that may affect their physical health and their ability to function normally. While clinical complications are assessed, postpartum functional outcomes are not well understood from a patient’s perspective or well-characterized by previous studies. In Rwanda, 11% of rural women deliver via c-section. This study explores the functional recovery of rural Rwandan women after c-section and assesses factors that predict poor functionality at postoperative day (POD) 30.

**Methods:**

Data were collected prospectively on POD 3, 11, and 30 from women delivering at Kirehe District Hospital between October 2019 and March 2020. Functionality was measured by self-reported overall health, energy level, mobility, self-care ability, and ability to perform usual activities; and each domain was rated on a 4-point likert scale, lower scores reflecting higher level of difficulties. Using the four functionality domains, we computed composite mean scores with a maximum score of 4.0 and we defined poor functionality as composite score of ≤ 2.0. We assessed functionality with descriptive statistics and logistic regression.

**Results:**

Of 617 patients, 54.0%, 25.9%, and 26.8% reported poor functional status at POD3, POD11, and POD30, respectively. At POD30, the most self-reported poor functionality dimensions were poor or very poor overall health (48.1%), and inability to perform usual activities (15.6%). In the adjusted model, women whose surgery lasted 30–45 min had higher odds of poor functionality (aOR = 1.85, *p* = 0.01), as did women who experienced intraoperative complications (aOR = 4.12, 95% CI (1.09, 25.57), *p* = 0.037). High income patients had incrementally lower significant odds of poor physical functionality (aOR = 0.62 for every US$1 increase in monthly income, 95% CI (0.40, 0.96) *p* = 0.04).

**Conclusion:**

We found a high proportion of poor physical functionality 30 days post-c-section in this Rwandan cohort. Surgery lasting > 30 min and intra-operative complications were associated with poor functionality, whereas a reported higher income status was associated with lower odds of poor functionality. Functional status assessments, monitoring and support should be included in post-partum care for women who delivered via c-section. Effective risk mitigating intervention should be implemented to recover functionality after c-section, particularly among low-income women and those undergoing longer surgical procedures or those with intraoperative complications.

## Introduction

When medically indicated, cesarean sections (c-sections) can be lifesaving for both mother and baby (WHO 2015). C-sections are the most common surgical procedures performed among women and in most LMIC facilities, the most common surgical procedure performed [1]. Furthermore, recent years have seen a rapid increase in c-section delivery rates, both in high-income and low- to middle-income countries (LMICs) [2, 3]. C-section delivery itself is associated with short- and long-term changes in a woman’s physical health, including altered functional status i.e. ability to carry out daily activities of life [4]. Recovery after c-section may have a lengthy trajectory [5] and better understanding of postpartum functional outcomes is key to informing clinical management and designing interventions that can optimize postpartum wellbeing.

As the backbone of households, many women are expected to maintain their duties, including day-to-day household management, care for their new infants and other children, marital and social relationships, or participate in community activities within a short period after childbirth [5, 6]. In rural and resource-constrained settings, particularly where large proportions of the population are agrarian or subsistence farmers [7, 8]; this context may also necessitate women to return to strenuous work shortly after childbirth [9, 10]. Functional recovery and resumption of routine activities after c-section, if not addressed, could have a long-term effect on a woman’s physical and emotional health, as well as have implications on the financial impact on a family after childbirth.

Currently, there is limited empirical evidence that describes functional recovery after c-sections, particularly in sub-Saharan Africa [11]. The available literature on post-c-section recovery focusses on postoperative clinical signs and symptoms complications, with little attention on functional abilities and the general wellbeing of women. However, women delivering via C-section even without complication may face physical discomfort for weeks, which may limit their ability to function in their daily lives and mobility [12, 13]. This has been demonstrated in studies from Sudan, China and India, where women that pain after c-section affected their ability to care for their newborns and problems related to mobility, self-care, or ability to perform usual activities through the first postoperative month [14, 15]. While there are no reported risk factors for poor post-c-section functional status, parity, duration of surgery, and preoperative depression have been shown to predict persistence of pain and to negatively affect recovery [16, 17].

In Rwanda, 17% of women nationally and 11% of rural women deliver via c-section (National Institute of Statistics of Rwanda (NISR) [Rwanda], Ministry of Health (MOH) [Rwanda] and ICF & International, 2015) [18]. While some studies have reported high rates of surgical site infections (SSIs) after c-section, ranging from 3%-17% [19–22], there is paucity of data assessing functional recovery among women who undergo c-section in rural Rwanda. This study reports functional status and predictors of poor functionality among rural Rwandan women through the first month post-cesarean delivery.

## Methods

This study was conducted as part of a large prospective cohort study designed to evaluate the feasibility, acceptability, and diagnostic efficacy of photo-enhanced screening for SSI diagnosis following cesarean delivery, and included data collected on c-section patients at three different time points: day of discharge (approximately post-operative day (POD) 3), POD 11 (± 3 days), and POD 30 (± 1 day).

### Setting

The parent study was conducted at Kirehe District Hospital (KDH), located in the Eastern Province of Rwanda. KDH encompasses 19 health centers and serves a population of over 360,000. Most Kirehe residents are insured through “*Mutuelle de Sante”—*a community-based health insurance scheme by which patients only pay 10% of their healthcare cost. KDH is managed by the Rwandan Ministry of Health (MoH) and receives technical and financial support from Partners In Health/Inshuti Mu Buzima (PIH/IMB), a Boston-based non-governmental international organization. In Kirehe, like other rural areas of Rwanda, labor is monitored at the local health center for normal delivery, while complicated deliveries, high-risk pregnancies, and women who need a c-section delivery are subsequently transferred to KDH for higher level management. C-sections at KDH are performed by general practitioners (GPs) and post-cesarean recovery is monitored by midwives or nurses. There is one obstetrician-gynecologist who assists GPs on more complicated cases and mentors them in the performance of emergency interventions. When there are no complications, a woman is hospitalized for three days after her c-section and is then discharged home to continue wound dressing changes and evaluation at her local health center. The standard procedures for C-section operation at KDH include:1) pre-operative intravenous injection of 2 mg of antibiotics (usually ampicillin or ceftriaxone) within one hour of the operation; 2) spinal analgesia; 3) skin preparation with chlorhexidine followed by Povidone iodine solution; 4) Pfannenstiel incision for the majority(over 70%) of cases and Cohens of midline incision if prior c-section used these techniques; 5)while bladder flap is recommended, it is not usually done by most doctors at KDH; 5) closure of the uterus with a double-layer, non-locking absorbent monofilament suture ( peritoneum is not usually sutured and the rectus muscle is sutured only when damaged); 6) skin closure with a continuous subcutaneous suture; 7) postoperative intramuscular injection of 75 mg of Diclofenac immediately after the operation and prescription of paracetamol or Ibuprofen 500 mg twice a day for 5 days, post-operatively. Post-operative antibiotic is provided on a case-by case basis.

### Study population

**Fig. 1:**
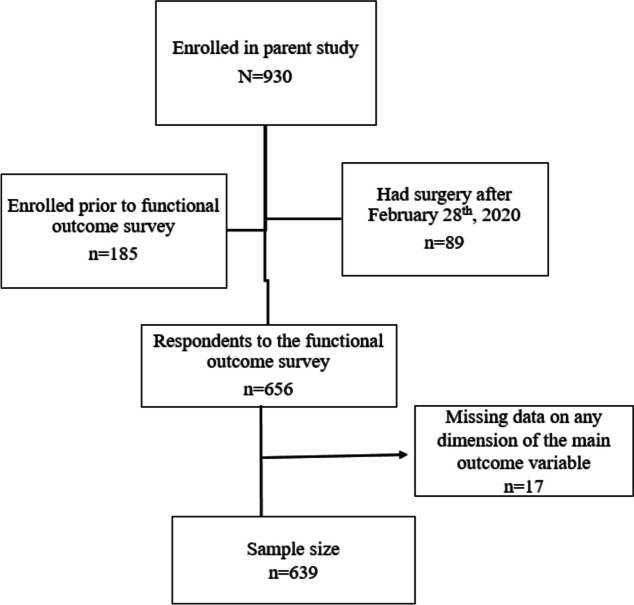
Flowchart of partcipants

The parent study recruited women, regardless of age, who were permanently residing in Kirehe District and who had a c-section delivery at KDH between September 23^rd^, 2019 and February 28^th^, 2020. In this analysis, we excluded women who consented to the study before the start of data collection on functional outcome variables (October 23^rd^, 2019), women who enrolled after February 28^th^, 2020 as we were unable to complete follow-up due to COVID-19 restrictions, and participants missing functional data at all three data collection points (Fig. 1). The parent study excluded women who resided outside the KDH’s catchment area and women from Mahama Refugee Camp.

### Primary data source and validation

At POD 1, experienced data collectors recruited patients, obtained consent, and administered a structured questionnaire to collect self-reported demographic data, including *Ubudehe* categories. *Ubudehe* is a four-tiered socioeconomic rank system, with *Ubudehe* 1 being the poorest rank and 4 being the wealthiest [23]. At the time of discharge (approximately POD 3), data collectors administered self-report questions on functional status and extracted clinical data from patients’ charts. Clinical elements collected in this study were based on data variables that were deemed to affect c-section outcomes. We administered a second survey on functional status at POD 11 (± 3 days), when participants had returned to the outpatient clinic for follow-up and physical exam. At POD 30, study team members called mothers and administered a third functional status survey.

Physical functional outcome as the main dependent variable in this study was defined using five dimensions: ability to move independently, ability to selfcare, ability to perform usual activities, level of energy, and overall health. We developed a 5 question interview addressing these dimensions with reference to the two previously validated tools: the World Health Disability Assessment Schedules (WHODAS 2.0) and the European Health-Related Quality of Life (EQ-5D) survey [24, 25]. We considered each of these standalone tools but due to length (WHODAS 2.0), and available resources and timeline needed for formal translation (EQ-5D) the decision was made not to use these tools. We used these tools to identify and adapt the targeted questions most relevant and feasible to administer to our study populations [25–27]. All five dimensions of the outcome variables were self-reported and included four levels, with 1 representing poorest or lowest level of functionality for each dimension and 4 representing the best or highest level of functionality.

At each time point, data collectors entered data into a password protected electronic data collection application (REDCap) [28, 29]. The study team lead regularly checked and confirmed the data quality. To maximize the response rate for the POD 30 phone call, we requested patients to provide multiple phone numbers (home phone, family member’s phone, and neighbor’s phone) on which they could be reached. We contacted a local community health worker to help trace participants who could not be reached on designated phone numbers. If participants could not be reached on the first attempt, they were called two additional times on two different days. The lost to follow up were those who could not be traced via their phone numbers of through a CHW.

### Analysis and statistics

We restricted data analysis to participants with data collected on at least one of the follow up time points. We summarized demographic and clinical characteristics of participants using frequencies and percentages for categorical data, and medians and interquartile ranges (IQR) for asymmetrically distributed continuous data. We described the five dimensions of functional status at POD 3, POD 11, and POD 30, using frequencies and percentages. We assessed functional status using a composite variable based on the mean scores of the five dimensions. Composite scores ranged from one to four with one representing the poorest level of functionality and 4 representing the highest level of functionality. We further dichotomized functionality into poor and good function, with scores of 2 or lower reflecting poor function and scores of greater than 2 representing good function. We assessed the association between demographic and clinical characteristics and poor functionality at POD 30 using Chi-square tests for categorical independent variables and Wilcoxon rank sum tests for continuous independent variables. Variables with a *p*-value < 0.2 in a bivariate analysis were considered for a multivariate analysis using logistic regression and were included in the full model. Subsequent reduced models were built using backward stepwise selection by which the least statistically significant variables were eliminated one at the time. We assessed statistical significance in the multivariate analysis at the *p*-value = 0.05 significance level. We used Stata/IC version 15.1 (College Station, TX: StataCorp LP) for all statistical analyses.

## Results

A total of 639 women were included in the study, with 511 (80.0%) having follow up at all three time points, 548 (85.8%) with follow up at two time points, and 602 (94.2%) with only one follow up. Most women were between 20 and 34 years old (*n* = 477, 74.6%), with 56 (8.8%) under 20 years and 106 (16.6) over 34 years (Table 1). The majority of women lived with a partner (*n* = 536, 83.9%). Most (*n* = 543, 85.0%) were farmers and the median self-reported annual household income was US$1,624 ($1,133, $2,675), with 72 participants (11.3%) belonging to the lowest rank of *Ubudehe* categories. The median travel time from the woman’s home to the health center was 30 min (IQR: 15, 60) and 40 min (IQR: 5, 60) from health center to the hospital. Primipara accounted for 40.4% (*n* = 258) of women, and 264 women (41.4%) had one or two previous births. Approximately 30% of women (*n* = 188) had a prior c-section delivery.

**Table 1:** Demographic and clinical characteristics of women delivering via c-section at Kirehe District Hospital

Variable name	Frequency (n)	Percentage (%)
**N**	639	100%
**Demographic Characteristics**		
**Age, in years**		
< 20	56	8.8%
20–24	191	29.9%
25–29	175	27.4%
30–34	111	17.4%
> = 35	106	16.6%
**Parity**		
0	258	40.4%
1–2	264	41.4%
3–4	73	11.4%
> 4	43	6.7%
**Marital Status**		
Single, separated, divorced/widow	103	16.1%
Legally married	237	37.1%
Cohabitating (common marriage)	299	46.8%
**Level of education**		
Less than primary	60	9.4%
Completed primary	431	67.5%
Completed secondary or more	148	23.1%
**Has health insurance**	636	99.5%
**Ubudehe categories**		
Ubudehe 1	72	11.3%
Ubudehe 2	334	52.4%
Ubudehe 3&4	231	36.3%
**Occupation**		
Farmer	543	85.0%
Employed (self, government, etc.)	74	11.6%
Unemployed (including housewives and students)	22	3.4%
**Travel time in minutes, home to health center, Median (IQR)**	30	(15,60)
**Travel time in minutes, health center to Hospital, Median (IQR)**	40	(5,60)
**Annual household Income in USD, Median (IQR)**^d^	$1,624	($1,133, $2675)
**Clinical Characteristics**		
**Had previous c-section**	188	29.66%
**Reasons for surgery (*N* = 630)**		
Fetal factors, no maternal factors^b^	213	33.8%
Maternal factors, no neonatal factors^a^	177	28.1%
Previous c-section only	140	22.2%
Maternal and fetal factors	100	15.9%
**Any intraoperative complication**^c^		
Yes	12	1.9%
Not documented	627	98.1%
**Duration of c-section surgery (in minutes) (*N* = 623)**		
< = 30	189	30.3%
(30–45)	290	46.5%
(45–60)	79	12.77%
> 60	65	10.4%
**Length of hospital stay post-c-section, in days (*N* = 601)**		
< = 3 days	240	39.9%
> 3 days	361	60.1%
**Surgical c-section categories**		
Emergent	38	6.0%
Urgent	562	88.1%
Elective	38	6.0%
**Neonatal outcomes at discharge (*N* = 631)**		
Alive and discharged	553	87.6%
Alive and admitted to NICU	63	10.0%
Died	15	2.4%

^a^Defined by any of the following: prior uterine scar, obstructed labor, contracted pelvis, hypertension, fever, hemorrhage, uterine rupture, placental abnormality

^b^Defined by any of the following: Fetal distress, mal presentation, malposition, macrosomia, multiple pregnancies, cord prolapse, intrauterine growth restriction

^c^Intraoperative complications include: Bleeding that requires transfusion, organ injury and uterine issues and difficult fetal extraction

^d^Converted using Rwanda purchasing power parity (PPP) for 2018

Nearly all women (*n* = 600, 94.1%) had an urgent or emergency surgery. The indications for c-section were: fetal factors only (*n* = 213 (33.8%), maternal factors only (*n* = 177, 28.1%), prior c-section (*n* = 140, 22%) and combined fetal and maternal factors (*n* = 100, 15.9%). Nearly half of all women (*n* = 290, 46.5%) had surgery length ranging between 30 to 45 min, 189 (30.3%) had a surgery length of less than 30 min, and 65 (10.4%) had a surgery length of greater than an hour. Over 60% (*n* = 361) of participants had a hospital stay greater than three days (Table 1).

Table 2 summarizes the five dimensions of functional status of patients at POD 3, POD 11 and POD 30. The majority of women were able to move independently at the time of hospital discharge (*n* = 518 of 622, 83.3%), at POD 11 (*n* = 499 of 589, 84.7%) and at POD30 (*n* = 500 of 549, 91.0%). Participants reported the lowest level of functionality in the dimension assessing ability to perform usual activities, with 50.8% (*n* = 316 of 622) of women reporting total inability to perform usual activities at hospital discharge and 15.6% (*n* = 86 of 551) at POD 30. At POD 30, 368 (out of 551, 68.6%) women reported needing help to perform usual activities, 268 (out of 551, 48.6%) reported poor or very poor overall health status, and 80 (14.5%) reported needing help to self-care. The functional status was poor for 54.0% (*n* = 333 of 617) and 26.8% (*n* = 147 of 548) of women at POD 3 and POD 30, respectively. Of the 147 women with poor functional status documented at POD 30, 78 (53%) had reported poor functional status at POD 3, while 79 (47%) had good functional status at POD 3 and deteriorated over the following month.

**Table 2:** Description of dimensions used to define physical functional status of women at Kirehe district hospital at hospital discharge and Post-operative days (POD)11 and POD 30

	**At discharge**		**POD11**		**POD30**	
**Dimension Of Function**	**n**	**%**	**n**	**%**	**n**	%
**Mobility/ Ability to move**	***N* = 622**		***N* = 589**		***N* = 549**	
I cannot move	1	0.2%	0	0.0%	2	0.4%
I need some help to move	30	4.8%	9	1.5%	28	5.1%
I need very little help to move	73	11.7%	81	13.8%	19	3.5%
I can move independently	518	83.3%	499	84.7%	500	91.0%
**Level of Energy**	***N* = 622**		***N* = 590**		***N* = 551**	
Very low	11	1.8%	12	2.1%	21	3.8%
Low	175	28.1%	114	19.3%	107	19.4%
Moderate	421	67.7%	412	69.8%	364	66.1%
High	15	2.4%	52	8.8%	59	10.7%
**Ability to perform usual activities**	***N* = 622**		***N* = 588**		***N* = 551**	
I cannot perform activities	316	50.8%	145	24.7%	86	15.6%
I need some help to perform activities	229	36.8%	165	28.1%	139	25.2%
I need little help performing activities	63	10.1%	148	25.2%	239	43.4%
I don’t need help	14	2.3%	130	22.1%	87	15.8%
**Ability to self-care**	***N* = 620**		***N* = 590**		***N* = 550**	
I cannot care for myself	11	1.8%	1	0.2%	8	1.5%
I need some help to care for myself	63	10.2%	14	2.4%	48	8.7%
I need little help care for myself	151	24.3%	82	13.9%	32	5.8%
I don’t need help to care for myself	395	63.7%	493	83.6%	462	84.0%
**Overall health status**	***N* = 623**		***N* = 590**		***N* = 551**	
Very poor	3	0.5%	3	0.5%	3	0.5%
Poor	394	63.2%	228	38.6%	265	48.1%
Good	218	35.0%	341	57.8%	242	43.9%
Very Good	8	1.3%	18	3.1%	41	7.4%
**Main Outcome**						
**Overall Functional Status**^a^	***N* = 617**		***N* = 587**		***N* = 548**	
Poor	333	54.0%	152	25.9%	147	26.8%
Good	284	46.0%	435	74.1%	401	73.2%

^a^Overall function status is a composite variable created by combining the above individual domains; scores 1–4 are generated and dichotomized into poor function status:1–2, good function satus:3–4, inclusive

Women who stayed in the hospital longer than three days after delivery were more likely to report poor functional status at POD 30 compared to those who stayed three days or fewer (*p* = 0.024, Table 3).

**Table 3:** Bivariate analysis of characteristics associated with poor physical functional status at Post-Operative Day (POD) 30

**Variable name**		**Poor function at POD30POD**		*P*-value^*^
	**N**	**n**	**%**	
**Number with poor functional outcome, overall**	548	147	26.8%	
**Demographic Characteristics**				
**Age, in years**				0.25
< 20	43	7	16.3%	
20–24	158	43	27.2%	
25–29	153	36	23.5%	
30–34	99	31	31.3%	
> = 35	95	30	31.6%	
**Parity (*N* = 547)**				0.43
0	217	51	23.5%	
1–2	229	64	28.0%	
3–4	63	19	30.2%	
> 4	38	13	34.2%	
**Marital Status**				0.80
Single, separated, divorced/widow	80	19	23.8%	
Legally married	231	58	27.5%	
Cohabitating (common marriage)	255	70	27.5%	
**Level of education**				0.13
Less than primary	54	13	24.1%	
Completed primary	359	106	29.5%	
Completed secondary or more	135	28	20.7%	
**Has health insurance**				0.18
No	3	2	66.7%	
Yes	545	145	26.1%	
***Ubudehe* categories**				0.47
*Ubudehe* 1	55	11	20.0%	
*Ubudehe* 2	289	80	27.7%	
*Ubudehe* 3&4	202	56	27.7%	
**Occupation**				0.09
Farmer	463	129	27.9%	
Employed (self, government, etc.)	67	17	25.4%	
Unemployed	18	1	5.6%	
**Travel time in minutes, home to health center, Median (IQR) (*N* = 579)**				0.18
Among those with poor function	134	30	(15,60)	
Among those with good function	361	30	(15,60)	
**Travel time in minutes, health center to hospital, Median (IQR) (*N* = 579)**				0.10
Among those with poor function	134	45	(10,60)	
Among those with good function	360	37.5	(5,60)	
**Monthly household Income in USD, Median (IQR)**^c^				
Among those with poor function	147	37.6	(28.3,62.4)	0.05
Among those with good function	370	45.4	(30.3,72.8)	
**Clinical Characteristics**				
**Had previous c-section (*N* = 636)**	162	43	29.45%	0.95
**Reasons for surgery (*N* = 630)**				0.28
Fetal factors, no maternal factors^a^	182	52	35.5%	
Maternal factors, no neonatal factors^b^	154	33	22.6%	
Previous c-section only	119	33	22.6%	
Maternal and fetal factors	87	28	19.2%	
**Duration of c-section surgery (in minutes)**	534			0.07
< = 30	163	33	20.2%	
(30–45)	247	78	31.6%	
(45–60)	70	20	28.6%	
> 60	54	12	22.2%	
**Any intraoperative complication**				0.10
Not reported	538	142	26.4%	
Yes	10	5	50%	
**Length of hospital stay, in days**	534			0.024
< = 3 days	163	33	20.3%	
> 3 days	371	110	29.7%	
**Surgical c-section categories**	547			0.74
Emergent	32	9	28.1%	
Urgent	481	127	26.4%	
Elective	34	11	32.4%	
**Neonatal outcomes at discharge**	542			0.31
Alive and discharged	487	127	26.1%	
Alive and admitted to NICU	41	15	36.6%	
Died	14	3	21.4%	

^*^Defined by any of the following: prior uterine scar, obstructed labor, contracted pelvis, hypertension, fever, hemorrhage, uterine rupture, placental abnormality

^a^Defined by any of the following: Fetal distress, mal presentation, malposition, macrosomia, multiple pregnancies, cord prolapse, intrauterine growth restriction

^b^Intraoperative complications include: Bleeding that requires transfusion, organ injury and uterine issues and difficult fetal extraction

^c^Converted using Rwanda purchasing power parity (PPP) for 2018

Those with a poor functional status had a significantly lower monthly income [poor functional status: $37.6, IQR: ($28.3, $62.4); good functional status: $45.4, IQR: ($30.3, $70.6); *p* = 0.04)]. In the final reduced model (Table 4), surgery duration, intraoperative complications, and income remained the only significant factors. Patients whose surgery duration ranged between 30 to 45 min had 85% increased odds of reporting poor functional status at POD 30 compared to those with surgery duration of less than 30 min (aOR = 1.85, 95% CI: 1.15, 2.95). Women with any documented intraoperative complications had 3.12 times higher odds of poor function compared to women without complications (aOR = 4.12, 95% CI: 1.09, 15.57). For every US$100 increase in monthly income, there was a 38% decrease in the odds of reporting poor functional status at POD 30 (aOR = 0.62, 95% CI: 0.40, 0.96).

**Table 4:** Multivariate analysis of characteristics independently associated with poor physical functional status at POD30

**Variable name**	**Full Model**			**Reduced Model**		
	**OR**	**95% CI**	***p*-value**	**OR**	**95% CI**	***p*-value**
**Level of education**						
Less than primary	**Ref**					
Completed primary	1.3	(0.64, 2.72)	0.44			
Completed secondary or more	0.9	(0.38, 2.11)	0.80			
**Has health insurance**						
No	**Ref**					
Yes	0.19	(0.02,2.19)	0.18			
**Occupation**						
Farmer	**Ref**					
Employed (self, gov., etc.)	1.2	(0.60,2.50)	0.58			
Unemployed	0.28	(0.03,2.21)	0.23			
**Travel time in minutes, home to health center, Median (IQR) (*N* = 579)**	0.99	(0.95, 1.04)	0.87			
**Travel time in minutes, Health center to hospital, Median (IQR) (*N* = 579)**	1.02	(0.99,1.04)	0.21			
**Monthly household income in USD, Median (IQR)**^b^	0.67	(0.42,1,06)	0.10	0.62	(0.40,0.96)	0.033
**Any intraoperative complication**^a^						
Not reported	**Ref**					
Yes	5.39	(1.29,22.53)	0.021	4.12	(1.09,25.57)	0.037
**Duration of c-section surgery (in minutes)**						
< = 30	**Ref**					
(30–45)	1.7	**(1.05,2.80)**	0.03	1.85	(1.15,2.95)	0.01
(45–60)	1.5	(0.76,2.91)	0.242	1.56	(0.82,2.99)	0.18
> 60	0.80	(0.35,1.82)	0.60	0.92	(0.42,2.03)	0.84
< = 3 days	**Ref**					
> 3 days	0.76	(0.50,1.15)	0.20			

^a^Intraoperative complications include: Bleeding that requires transfusion, organ injury and uterine issues and difficult fetal extraction

^b^Converted using Rwanda purchasing power parity (PPP) for 2018

## Discussion

Our study is one of a few assessing the overall functional status of c-section patients a month after surgery in LMICs. We found a high proportion of poor functionality at discharge, which was sustained through the first month, with approximately one in four women reporting poor function at POD 30. Surgery duration of between 30 to 45 min, report of any intraoperative complications and lower annual household income were significant independent predictors of poor physical functional status at POD30.

Our rates are comparable to one study including women from Malawi, Kenya, and Jamaica, where 18% of women had poor function at six weeks postpartum [30]; however notably, our studies used different tools to measure physical functionality and included women despite the report of complications. For example, a study in Iran measured functionality as a single dimension of the health-related quality of life questionnaires, excluded women with complicated child birth or with poor neonatal outcomes, and reported an average functionality score of 53% at 2 months post-cesarean, with higher scores reflecting better physical functioning [31].

In our study, mobility was the fastest dimension to improve after c-section, with over 83% of women reporting full mobility at POD 3 and rising to 91% at POD 30. This aligns with findings from other LMICs where 93% to 98% of c-section patients reported good mobility through the first month following surgery [15, 32]. Despite high mobility, women reported poor ability to perform usual activities, with only 16% of women returning to full abilities by POD 30. Similarly other studies have demonstrated that tiredness and fatigue are common post-c-section and may impede early resumption of activities in these patients [33, 34]. However, it is also possible that some activities, including farming activities or intensive housework such as carrying water or older children, should not be immediately resumed as extreme physical activity or heavy lifting may impede safe recovery after cesarean delivery [35]. Unfortunately, rural lifestyle and limited family assistance can impose heavy work on women, even when they still have low abilities to perform activities. In rural Rwanda, women reported that family support with activities subsided after the first week of delivery; thereafter women were exposed to heavy workload [36]. Similarly in rural South Africa, women reported that even though they had planned to take longer time off, they had to engage in paid work, in addition to home and child care activities within the first two weeks of surgery, as aa result of limited assistance at home [37]. Such early exposure to increased workload may exacerbate fatigue and slow down timely recovery after c-section.

We found that women with a longer surgery duration and intraoperative complications were more likely to report poor physical function compared to shorter and non-complicated surgery. Surgery length has previously been shown to varies by health providers and by intraoperative complications [38–41]. Despite controlling for intraoperative complication, it is possible that surgeries with longer duration represented more complex cases of women with pre-existing medical co-morbidities, and it is these unmeasured factors that impacted recovery, rather than the surgery length itself i.e. there are other unmeasured confounding factors [42–45]. For example, the existing pathway in Rwanda requires laboring women to present at health centers before being referred to district hospitals for a C-section delivery (when indicated) could result in further complexities of cases during transportation to district hospitals and inherent delays [46]. In our study, 94.1% of c-section were indicated as emergent or urgent. These complex pregnancies and exacerbated labor anomalies in of themselves may be related to the course of postoperative recovery we documented, rather than the surgery length. Improvement in referral systems and decentralizing obstetric emergency services to health centers to enhance timely access to c-sections for medically indicated cases could result in fewer complications and unnecessarily long c-section surgeries potentially resulting in improved return to normal function [47, 48].

Women with lower annual household incomes had worse physical functional outcomes at POD 30. This association could be explained in the context of poor health outcomes previously reported among poor postpartum women. Poverty has been associated with poor postpartum nutritional status [49, 50], incidence of SSI [22, 51] and poor post-natal care [52]. A study in Ethiopia reported that irrespective of mode of delivery, women with low income were almost half as likely to seek postpartum care [53], and a study in Iran found that rural women who did not receive enhanced postpartum follow-up reported poor functional status at six weeks postpartum [54]. Financial hardship faced by poor women in our study could affect post-discharge follow-up and care, and in turn could impede their ability to recover fully. Moreover, financial stress in low-income households are known to elevate the risk of postpartum depression [55, 56], which also affects postoperative recovery [57, 58]. Thus, interventions to mitigate financial hardships should be explored in the future.

Our findings has several implications. We found that one in four women reported poor functional outcomes at POD 30. Developing strategies to improve the functional recovery for women after c-section could thus have substantial impact on improving physical and emotional wellbeing for families in the postpartum period, as well as potentially mitigate the financial impact of childbirth by c-section on families. Indeed in this same cohort we have previously reported that 6.4% and 27% of women experienced impoverishing and catastrophic expenditures, respectively due to hospital expenses alone, without consideration of the impact of lost economic productivity [59]. Both upstream and downstream strategies should be considered to help aid in the return to normal functional during the postpartum period. For example, increasing evidence points to changes in pre-operative and post-operative management, such as enhanced recovery after surgery (ERAS), leading to significant reductions in length of post-operative stay and time to mobility [60, 61]. However, evidence to date suggests ERAS procedures have yet to be implemented in many resource-limited settings. In Rwanda specifically, existing and well-established systems such as antenatal care clinics at health centers and community health worker systems could be explored as additional channels for implementing components ERAS, alongside clinical teams at district hospitals where surgeries are performed.

We note a number of limitations of this study. First, there was no available validated measurement for functional status for women delivered via c-section; nevertheless, we used individual dimensions pulled from different validated health-related quality of life tools to build a multifaceted measure of functionality. As noted, our choice to use this measure improved study feasibility however, this has limitations around comparing our data to other studies using available tools such as the WHODAS or EQ-5. Secondly, some recruited participants (8.6%) could not be reached by phone on POD 30, although several attempts were made. Loss-to-follow-up is possibly associated with the poorest women, who have limited access to a telephone, or those with postoperative morbidities seeking care outside their community or preventing access to or use of a phone, including but not limited to postpartum depression. As such we may have underestimated the level of poor functional status at POD 30. Thirdly, we did not have a control group to ascertain whether the reported functional status was attributable to c-section specifically, or could be attributed to childbirth more generally, and challenges around child care in the postpartum period. Fourth, there was no data on physical functional status before c-section which could have been useful in estimating the toll of c-section delivery on functionality and what constitutes return to baseline levels of functional status. Fifth, our last point of follow up was at POD30, which may represent a time frame too early in the post-operative period to expect return to normal function. Further studies assessing function further out from surgery will be needed to assess for the long term implications of c-section and its indications on the trajectory of functional recovery. Sixth, our functional outcome tool did not include aspects of postpartum mental health. Thus, our estimate of functional outcome may be underestimate given the potential influence postpartum depression may have on postpartum functional recovery. Further research should explore the development and validation of a concise functional recovery tool specific for C-section patients, and these tools should include the dimension of postpartum depression. Such development could include testing and validation against larger and more robustly used tools such as the WHODAS Lastly, it is possible that women did not have clarity of which activities they should resume or not resume during the first month after surgery because in Rwanda, currently there is no educational protocol to inform c-section women with their post-cesarean recovery. However, we believe that this did not severely affect our results because functionality was measured as a multidimensional outcome.

## Conclusion

The present study provides evidence that a quarter of rural Rwandan women sustain poor physical functional status up to a month after cesarean delivery. Particularly, women from low income households, women who experience intraoperative complications, and women with longer surgeries were more vulnerable to poor functionality. Specific attention should be paid to these women during follow-up. Functional status assessments, monitoring and support to functional recovery should be included in post-partum care. Guidelines for postpartum care providers and women should be developed and address physical aspects of functionality, including resumption of usual activities, physical activities and body energy during post-partum period. We believe that these findings hold significance in other rural LMIC settings. Given rising global c-section rates, future research is needed to develop and validate a comprehensive measure of post-cesarean functionality. Further research into functional outcomes after c-section in other LMIC settings should be considered as well as research into peri-operative interventions that may improve and speed up return to normal functionality.

## Data Availability

Data is available upon reasonable request from corresponding author.
